# Association of Urine Albumin to Creatinine Ratio With Cardiovascular Outcomes in Patients With Type 2 Diabetes Mellitus

**DOI:** 10.1210/clinem/dgad645

**Published:** 2023-10-31

**Authors:** Cheng Zeng, Maojun Liu, Yifeng Zhang, Simin Deng, Ying Xin, Xinqun Hu

**Affiliations:** Department of Cardiology, The Second Xiangya Hospital of Central South University, No. 139, Middle Ren-min Road, Changsha 410011, Hunan Province, People's Republic of China; Department of Cardiology, The Second Xiangya Hospital of Central South University, No. 139, Middle Ren-min Road, Changsha 410011, Hunan Province, People's Republic of China; Department of Cardiology, The Second Xiangya Hospital of Central South University, No. 139, Middle Ren-min Road, Changsha 410011, Hunan Province, People's Republic of China; Department of Cardiology, The Second Xiangya Hospital of Central South University, No. 139, Middle Ren-min Road, Changsha 410011, Hunan Province, People's Republic of China; Department of Cardiology, The Second Xiangya Hospital of Central South University, No. 139, Middle Ren-min Road, Changsha 410011, Hunan Province, People's Republic of China; Department of Cardiology, The Second Xiangya Hospital of Central South University, No. 139, Middle Ren-min Road, Changsha 410011, Hunan Province, People's Republic of China

**Keywords:** major adverse cardiovascular events, total mortality, type 2 diabetes mellitus, urinary albumin to creatinine ratio

## Abstract

**Context:**

The urinary albumin to creatinine ratio (UACR) is a widely used indicator of albuminuria and has predictive value for adverse cardiovascular events.

**Objective:**

To evaluate the correlation between the UACR and the risk of developing major adverse cardiovascular events (MACEs) and total mortality in patients with type 2 diabetes mellitus (T2DM).

**Methods:**

This post hoc analysis included 10 171 participants from the Action to Control Cardiovascular Risk in Diabetes (ACCORD) study and the ACCORD follow-up study (ACCORDION) with baseline UACR data. The natural logarithm (ln) of each UACR measurement was calculated. Univariate and multivariate Cox proportional hazard regression analyses were conducted to examine the association between the UACR and the risk of MACEs and total mortality. The additional predictive value of UACR was further evaluated. Similar methods were used to analyze the correlation between the UACR and MACEs and total mortality within the normal range.

**Results:**

During a median follow-up period of 8.83 years, 1808 (17.78%) participants experienced MACEs, and there were 1934 (19.01%) total deaths. After adjusting for traditional cardiovascular risk factors, the multivariate analysis revealed a significant association between the UACR and the risk of MACEs and total mortality. The inclusion of UACR in the conventional risk model enhanced the predictive efficacy for MACEs and total mortality.

**Conclusion:**

An elevated UACR is associated with a higher risk of MACEs and total mortality in patients with T2DM, even when it falls within the normal range. The UACR improves prediction of MACE and total mortality risk in patients with T2DM.

The global prevalence of diabetes mellitus (DM) among people aged 20 to 79 years was estimated to be 10.5% in 2021, and this figure is expected to increase to 12.2% by 2024 (1). The global financial outlays attributed to diabetes-related health care were approximately 966 billion USD in 2021, with projections indicating an increase to 1054 billion USD by 2045 (1). Cardiovascular disease (CVD) is the primary cause of mortality and morbidity in patients with DM (2). Individuals with DM are at higher risk of major adverse cardiovascular events (MACEs) (3). Even with meticulous management of hyperglycemia, dyslipidemia, and hypertension in individuals with DM, the residual risk of diabetes persists considerably (4). Moreover, patients with diabetic kidney disease have an increased incidence of CVDs (5).

Albuminuria is a risk factor for both morbidity and mortality associated with CVD in patients with DM (6). The urinary albumin to creatinine ratio (UACR) is a common indicator of urinary albumin level and is associated with the incidence of cardiovascular events, particularly in individuals with DM, hypertension, or dyslipidemia (6-8). The UACR, a marker of early endothelial dysfunction, is associated with the presence of subclinical atherosclerosis (9, 10). For every 30% reduction in the UACR, the risk of cardiovascular death is reduced by 14% (11). Therefore, the UACR has a high predictive value for cardiovascular events in patients with DM.

The American Diabetes Association recommends maintaining UACR below 30 mg/g for patients with type 2 diabetes mellitus (T2DM) (7). However, several studies have indicated that the risk of MACEs and total mortality increased even when UACR was elevated within the normal range (<30 mg/g) (12-15). Limited research has been conducted on the influence of elevated UACR within normal ranges on adverse cardiovascular events in patients with T2DM.

This study aimed to explore the association between the UACR and the risk of MACEs and total mortality in patients with T2DM using data from the Action to Control Cardiovascular Risk in Diabetes (ACCORD) clinical trial and the ACCORD follow-up study (ACCORDION).

## Materials and Methods

### Study Design and Participants

We used data from the ACCORD/ACCORDION trial (ClinicalTrials.gov number: NCT00000620) for post hoc analysis. The rationale, design, and primary outcomes of the ACCORD study have been previously described and published (16, 17). In short, ACCORD was a randomized, multicenter, double 2 × 2 factorial trial involving 10 251 patients (mean age, 62.2 years; median glycated hemoglobin [HbA1c], 8.1%) with T2DM who were at high risk for developing a CVD. The patients were treated and followed up for an average of approximately 5 years from 2001 through mid-2009. The study was designed to investigate whether strict control of blood glucose levels, hypertension, and lipid levels can reduce the incidence of CVDs in patients with T2DM. ACCORD participants who agreed to participate in ACCORDION were followed up through clinic and phone visits for an average of 3.5 years from 2011 to 2014. This provided the ACCORDION participants with approximately 10 years of post-randomization follow-up.

### Data Collection and Outcomes

**Figure 1.**
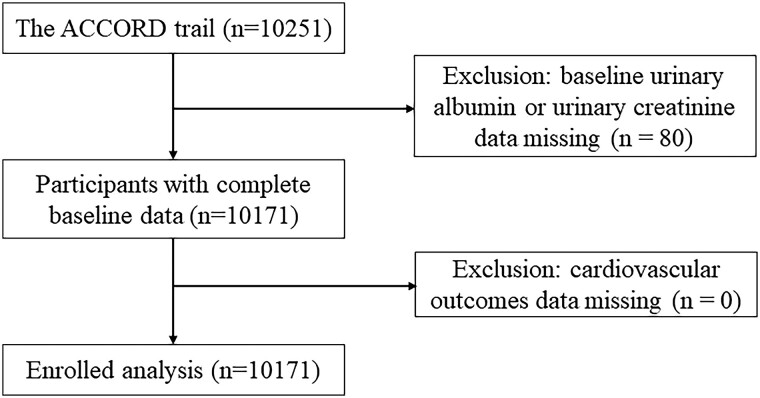
Flowchart of this study.

The data we used included demographic and clinical characteristics, age, sex, race, treatment, education, previous medical history, physical examination, laboratory examination (ie, estimated glomerular filtration rate [eGFR], and glycated hemoglobin [HbA1c], total cholesterol [TC], triglyceride [TG], very low-density lipoprotein [VLDL], low-density lipoprotein cholesterol [LDL-C], high-density lipoprotein cholesterol [HDL-C], alanine aminotransferase [ALT], serum creatinine, urinary albumin, and urinary creatinine levels), and previous medication history. Of the 10 251 participants, 80 had no urinary albumin or urinary creatinine data at baseline, and 10 171 participants were eventually included in this study (Fig. 1).

The primary outcome of this study was the occurrence of MACEs, including CVD mortality, nonfatal myocardial infarction (MI), and nonfatal stroke. The secondary outcome was total mortality.

### Statistical Analysis

Statistical analyses were conducted using SPSS 26.0 (IBM, Armonk, NY, USA), R (The R Foundation, Vienna, Austria), and EmpowerStats (X&Y Solutions, Inc., Boston, USA) software packages. To normalize the skewed distribution of the UACR, the natural logarithm (ln) of each measurement was calculated. This transformation helps to create a more symmetric distribution and reduces the impact of extreme values. Since the UACR value may be less than 1 (resulting in a negative ln value), it was multiplied by 100 prior to transformation.

Baseline characteristics are expressed as mean ± SD, frequency with percentage, or median and interquartile range, according to the distribution type. Continuous variables were compared using analysis of variance (ANOVA) or the Kruskal-Wallis test, while categorical variables were compared using chi-square analysis.

The cumulative hazard of MACEs and total mortality were calculated using the Kaplan-Meier method based on the ln (100 × UACR) quantile. The differences in estimates were compared using log-rank tests. We used a Cox proportional hazards regression model to compute the hazard ratios (HRs) and corresponding 95% CIs for MACEs. In preparation for the multivariate Cox regression analysis, we conducted a preliminary univariate analysis to assess the relationship between each variable and the occurrence of MACEs elsewhere (18). Variables with a significance level of *P* < .10 in the univariate analysis were included in the multivariate analysis. Variables closely related to MACEs, such as body mass index (BMI) and family history of heart disease, heart attack, or stroke, were also included in the multivariate analysis.

We used a multivariate model with 3 degrees of progressive adjustment to control for potential confounders of MACEs. In addition, we used the area under the receiver operating characteristic (ROC) area under the curve (AUC), net reclassification improvement (NRI), and integrated discrimination improvement (IDI) to evaluate the additional predictive value of UACR beyond conventional risk factors.

We conducted subgroup and interaction analyses stratified by sex, age (<65 years and ≥65 years), race, treatment group (standard glucose control and intensive glucose control), CVD history, heart failure, previous hyperlipidemia, previous hypertension, duration of diabetes (<10 years and ≥10 years), BMI (<25 kg/m^2^ and ≥25 kg/m^2^), HbA1c (<8.1% and ≥8.1%), eGFR (<60 mL/min/1.73 m^2^, ≥60 mL/min/1.73 m^2^, <90 mL/min/1.73 m^2^, and ≥90 mL/min/1.73 m^2^), and insulin use.

## Results

### Baseline Characteristics Stratified by Tertiles of ln (100 × UACR)

The 10 171 participants included in this study were divided into 3 groups based on ln (100 × UACR). Table 1 presents the baseline characteristics of the study population. Approximately 61.44% were male, the mean age was 62.81 ± 6.66 years, and approximately 62.46% were of White race. The tertile ranges of ln (100 × UACR) were low (4.27–6.73 mg/g), middle (6.73–7.88 mg/g), and high (7.88–14.07 mg/g), and the corresponding UACRs were low (0.72–8.33), middle (8.33–26.52), and high (26.52–12 908.16), respectively. Participants exhibiting elevated tertile levels of ln (100 × UACR) were found to have a higher propensity for being male; race other than White; having a CVD or heart failure history; having a family history of heart disease, heart attack, or stroke; being associated with previous hypertension, longer duration of diabetes, and proteinuria; possessing elevated BMI, systolic blood pressure (SBP), diastolic blood pressure (DBP), heart rate, HbA1c, TG, VLDL, and serum creatinine levels; possessing declining HDL-C, ALT, and eGFR levels; and using diuretics, ARBs/ACEIs, CCBs, beta-blockers, or insulins.

**Table 1.** Baseline characteristics of participants by tertiles of ln (100 × UACR)

Characteristics	Low (n = 3389)	Middle (n = 3391)	High (n = 3391)	*P* value
UACR, mg/g	5.45 (4.23–6.84)	13.60 (10.58–18.23)	85.28 (44.24–227.48)	<.001
Ln (100 × UACR), mg/g	6.26 (0.33)	7.24 (0.32)	9.33 (1.15)	
Age, years	62.18 (6.27)	62.87 (6.60)	63.38 (7.04)	<.001
Sex, n (%)				<.001
Male	2030 (59.90%)	1974 (58.21%)	2245 (66.20%)	
Female	1359 (40.10%)	1417 (41.79%)	1146 (33.80%)	
Race, n (%)				.010
White	2152 (63.50%)	2153 (63.49%)	2048 (60.40%)	
Non-White	1237 (36.50%)	1238 (36.51%)	1343 (39.60%)	
Treatment, n (%)				.744
Standard glucose control	1696 (50.04%)	1707 (50.34%)	1676 (49.42%)	
Intensive glucose control	1693 (49.96%)	1684 (49.66%)	1715 (50.58%)	
Education, n (%)				<.001
Less than high school graduate	439 (12.96%)	479 (14.13%)	580 (17.12%)	
High school grad (or GED)	845 (24.95%)	949 (27.99%)	897 (26.48%)	
Some college or technical school	1152 (34.01%)	1071 (31.59%)	1113 (32.86%)	
College graduate or more	951 (28.08%)	891 (26.28%)	797 (23.53%)	
Living alone, n (%)	2743 (80.99%)	2712 (79.98%)	2653 (78.24%)	.017
Depression, n (%)	827 (24.42%)	814 (24.00%)	763 (22.50%)	.148
CVD history, n (%)	1022 (30.16%)	1163 (34.30%)	1398 (41.23%)	<.001
Family history of heart disease, heart attack, or stroke, n (%)	1593 (48.72%)	1654 (50.53%)	1634 (50.05%)	.314
Heart failure, n (%)	124 (3.66%)	155 (4.57%)	211 (6.22%)	<.001
Previous hyperlipidemia, n (%)	2385 (70.37%)	2422 (71.42%)	2311 (68.15%)	.011
Previous hypertension, n (%)	2413 (71.20%)	2579 (76.05%)	2673 (78.83%)	<.001
Duration of diabetes (years)	9.60 (7.14)	10.48 (7.42)	12.32 (7.94)	<.001
Proteinuria, n (%)	304 (8.97%)	538 (15.87%)	1179 (34.77%)	<.001
BMI, kg/m^2^	32.07 (5.26)	32.15 (5.41)	32.45 (5.52)	.018
SBP, mmHg	131.56 (15.54)	135.56 (16.22)	141.95 (17.86)	<.001
DBP, mmHg	74.17 (9.99)	74.82 (10.77)	75.65 (11.13)	<.001
Heart rate, bpm	72.07 (11.32)	72.80 (11.65)	73.10 (12.24)	.002
HbA1C, %	8.11 (0.97)	8.31 (1.04)	8.48 (1.12)	<.001
TC, mg/dL	181.55 (39.36)	183.42 (41.02)	185.07 (44.84)	.069
TG, mg/dL	175.60 (124.00)	186.49 (132.06)	208.51 (181.09)	<.001
VLDL, mg/dL	34.08 (20.60)	36.08 (22.84)	39.54 (28.68)	<.001
LDL-C (mg/dL)	104.67 (32.90)	105.42 (33.48)	104.63 (35.28)	.384
HDL-C, mg/dL	42.81 (11.61)	41.92 (11.33)	40.91 (11.82)	<.001
ALT, mg/dL	27.83 (17.48)	27.83 (15.50)	27.14 (15.54)	<.001
Serum creatinine, mg/dL	0.90 (0.21)	0.89 (0.22)	0.95 (0.26)	<.001
eGFR, mL/min/1.73 m^2^	90.73 (23.20)	92.93 (27.57)	89.60 (30.21)	<.001
Urinary albumin, mg/dL	0.71 (0.43)	1.81 (1.19)	28.27 (59.41)	<.001
Urinary creatinine, g/dL	0.13 (0.07)	0.12 (0.07)	0.12 (0.07)	<.001
Medications, n (%)				
Diuretics	1162 (34.29%)	1204 (35.51%)	1356 (39.99%)	<.001
ARB/ACEI	2220 (65.51%)	2327 (68.62%)	2501 (73.75%)	<.001
CCB	512 (15.11%)	583 (17.19%)	857 (25.27%)	<.001
Beta-blockers	856 (25.30%)	1009 (29.85%)	1188 (35.14%)	<.001
Sulfonylureas	1840 (54.31%)	1836 (54.14%)	1753 (51.70%)	.054
Biguanides	2140 (63.16%)	2224 (65.59%)	2139 (63.08%)	.051
Meglitinides	92 (2.72%)	88 (2.60%)	75 (2.21%)	.383
Thiazolidinediones	772 (22.79%)	731 (21.56%)	738 (21.76%)	.425
Insulins	1020 (30.10%)	1131 (33.35%)	1405 (41.43%)	<.001
Statins	2142 (63.41%)	2175 (64.43%)	2135 (63.22%)	.542
Fibrates	204 (6.04%)	220 (6.53%)	199 (5.89%)	.529
Cholesterol absorption inhibitors	66 (1.95%)	72 (2.14%)	68 (2.01%)	.866
MACEs, n (%)	401 (11.83%)	569 (16.78%)	838 (24.71%)	<.001
CVD mortality	108 (3.19%)	194 (5.72%)	359 (10.59%)	<.001
Nonfatal MI	226 (6.67%)	287 (8.46%)	417 (12.30%)	<.001
Nonfatal stroke	109 (3.22%)	164 (4.84%)	210 (6.19%)	<.001
Total mortality, n (%)	395 (11.66%)	602 (17.75%)	937 (27.63%)	<.001

Data are shown as mean (SD), median (Q1-Q3), or as n (%). The tertile ranges of ln (100 × UACR) were low (4.27–6.73 mg/g), middle (6.73–7.88 mg/g), and high (7.88–14.07 mg/g). *P* values for the test of the difference across tertiles of ln (100 × UACR) were obtained by using the χ^2^ test (categorical variables), ANOVA (continuous variables), or Kruskal-Wallis test (nonparametric comparisons).

Abbreviations: ALT, alanine aminotransferase; ARB, angiotensin receptor blocker; ACEI, angiotensin converting enzyme inhibitors; BMI, body mass index; CCB, calcium channel blockers; CVD, cardiovascular disease; DBP, diastolic blood pressure; eGFR, estimated glomerular filtration rate; GED, General Equivalency Diploma; HDL-C, high-density lipoprotein cholesterol; LDL-C, low-density lipoprotein cholesterol; MACEs, major adverse cardiovascular events; MI, myocardial infarction; SBP, systolic blood pressure; TC, total cholesterol; TG, triglycerides; UACR, urinary albumin to creatinine ratio; VLDL, very low-density lipoprotein.

### The Relationship Between ln (100 × UACR) and Outcomes

Participants with a high tertile ln (100 × UACR) were at an increased risk of MACEs and total mortality (Table 1).

Among all participants, during a median follow-up period of 8.83 years, 1808 (17.78%) participants experienced MACEs, including 661 CVD deaths (6.5%), 930 (9.14%) who had a nonfatal MI, and 483 (4.75%) who had a nonfatal stroke. Overall, there were a total of 1934 (19.01%) deaths.

**Figure 2.**
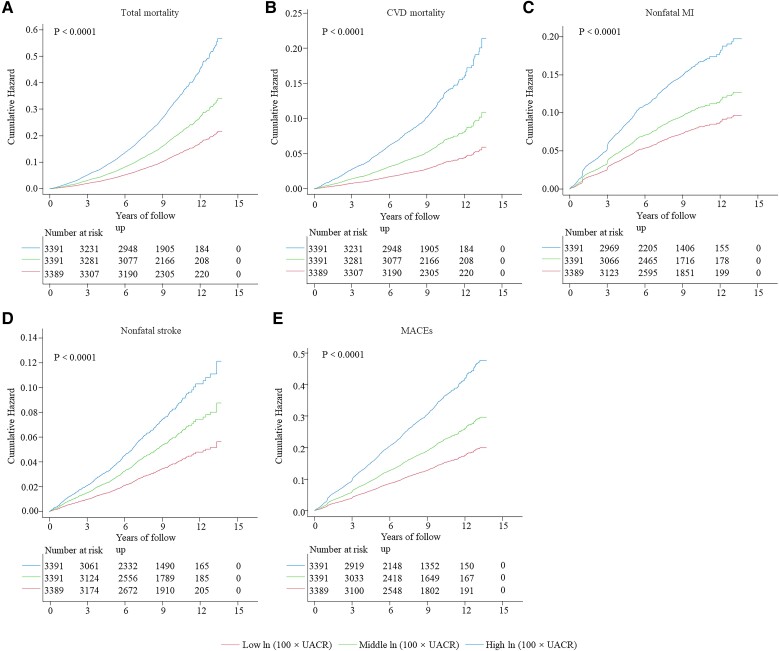
Kaplan-Meier curves for MACEs and total mortality. Middle and high ln (100 × UACR) vs low ln (100 × UACR). A, Total mortality; B, CVD mortality; C, Nonfatal MI; D, Nonfatal stroke; E, MACEs. Abbreviations: CVD, cardiovascular disease; MACEs, major adverse cardiovascular events; MI, myocardial infarction; UACR, urinary albumin to creatinine ratio.

Kaplan-Meier curves were used to assess the cumulative hazards of MACE (including CVD mortality, nonfatal MI, and nonfatal stroke) and total mortality (Fig. 2). The log-rank test revealed a statistically significant difference between the curves (*P* < .0001). Participants with elevated ln (100 × UACR) exhibited higher cumulative hazards than those with lower ln (100 × UACR).

Three multivariate regression models were used to examine the correlation between ln (100 × UACR) and the occurrence of outcome events (Table 2). Model 1 was adjusted for age, sex, race, treatment, education, living alone, depression, CVD history, family history of heart disease, heart attack, or stroke; heart failure history, previous hypertension, duration of diabetes, proteinuria, BMI, SBP, and DBP. Model 2 was further adjusted for HbA1c, TC, TG, VLDL, LDL-C, HDL-C, ALT, serum creatinine, and eGFR. Model 3 was adjusted for medication use (diuretics, ARBs/ACEIs, CCB, beta-blockers, sulfonylureas, biguanides, thiazolidinediones, and insulin) as an additional covariate for Model 2. In Model 3, the cumulative risk of MACEs demonstrated an increased association with ln (100 × UACR), even after comprehensive adjustment for potential confounding factors (middle HR 1.35; 95% CI, 1.18–1.54, *P* < .0001; high HR 1.78; 95% CI, 1.56–2.04, *P* < .0001). For each 1 SD increase in ln (100 × UACR), there was a 29% higher risk of participants developing MACEs (HR 1.29; 95% CI, 1.23–1.35, *P* < .0001). Moreover, the baseline ln (100 × UACR) remained significantly associated with CVD mortality, nonfatal MI, and nonfatal stroke outcomes. Additionally, the baseline ln (100 × UACR) also showed a significant association with total mortality (middle HR 1.42; 95% CI, 1.24–1.62, *P* < .0001; high HR 2.04; 95% CI, 1.79–2.32, *P* < .0001). For each 1 SD increase in ln (100 × UACR), participants had a 38% higher risk of total mortality (HR 1.38; 95% CI, 1.32–1.45, *P* < .0001). This finding suggests that ln (100 × UACR) can serve as a predictive factor for MACEs and total mortality in patients with T2DM.

**Table 2.** Risk of MACEs and total mortality based on ln (100 × UACR)

Outcome	Events/n	Non-adjusted		Model 1		Model 2		Model 3	
		HR (95% CI)	*P* value	HR (95% CI)	*P* value	HR (95% CI)	*P* value	HR (95% CI)	*P* value
**MACEs**									
Ln (100 × UACR)		1.28 (1.25, 1.32)	<.0001	1.22 (1.18, 1.26)	<.0001	1.19 (1.16, 1.24)	<.0001	1.19 (1.15, 1.23)	<.0001
Low	401/3389	Ref		Ref		Ref		Ref	
Middle	569/3391	1.48 (1.30, 1.68)	<.0001	1.39 (1.22, 1.59)	<.0001	1.35 (1.18, 1.54)	<.0001	1.35 (1.18, 1.54)	<.0001
High	838/3391	2.38 (2.11, 2.68)	<.0001	1.90 (1.67, 2.17)	<.0001	1.79 (1.56, 2.04)	<.0001	1.78 (1.56, 2.04)	<.0001
Per 1 SD		1.44 (1.38, 1.50)	<.0001	1.34 (1.27, 1.40)	<.0001	1.30 (1.24, 1.36)	<.0001	1.29 (1.23, 1.35)	<.0001
*P* for trend			<.0001		<.0001		<.0001		<.0001
**CVD mortality**									
Ln (100 × UACR)		1.41 (1.35, 1.47)	<.0001	1.36 (1.29, 1.43)	<.0001	1.33 (1.27, 1.41)	<.0001	1.33 (1.26, 1.40)	<.0001
Low	108/3389	Ref		Ref		Ref		Ref	
Middle	194/3391	1.85 (1.46, 2.34)	<.0001	1.70 (1.33, 2.16)	<.0001	1.66 (1.30, 2.12)	<.0001	1.66 (1.30, 2.12)	<.0001
High	359/3391	3.64 (2.94, 4.52)	<.0001	2.85 (2.26, 3.60)	<.0001	2.68 (2.11, 3.40)	<.0001	2.69 (2.11, 3.42)	<.0001
Per 1 SD		1.65 (1.55, 1.76)	<.0001	1.57 (1.46, 1.70)	<.0001	1.53 (1.41, 1.65)	<.0001	1.52 (1.41, 1.64)	<.0001
*P* for trend			<.0001		<.0001		<.0001		<.0001
**Nonfatal MI**									
Ln (100 × UACR)		1.24 (1.20, 1.29)	<.0001	1.20 (1.15, 1.26)	<.0001	1.18 (1.13, 1.24)	<.0001	1.17 (1.12, 1.23)	<.0001
Low	226/3389	Ref		Ref		Ref		Ref	
Middle	287/3391	1.31 (1.10, 1.56)	.0023	1.26 (1.06, 1.51)	.0109	1.23 (1.03, 1.48)	.0241	1.23 (1.02, 1.48)	.0261
High	417/3391	2.04 (1.74, 2.40)	<.0001	1.73 (1.45, 2.07)	<.0001	1.65 (1.37, 1.98)	<.0001	1.63 (1.35, 1.95)	<.0001
Per 1 SD		1.38 (1.30, 1.46)	<.0001	1.31 (1.22, 1.40)	<.0001	1.28 (1.19, 1.37)	<.0001	1.26 (1.18, 1.36)	<.0001
*P* for trend			<.0001		<.0001		<.0001		<.0001
**Nonfatal stroke**									
Ln (100 × UACR)		1.23 (1.16, 1.29)	<.0001	1.12 (1.05, 1.19)	.0007	1.09 (1.03, 1.17)	.0066	1.09 (1.02, 1.17)	.0072
Low	109/3389	Ref		Ref		Ref		Ref	
Middle	164/3391	1.56 (1.22, 1.98)	.0003	1.35 (1.06, 1.73)	.0165	1.29 (1.01, 1.66)	.0426	1.30 (1.01, 1.66)	.0415
High	210/3391	2.15 (1.71, 2.72)	<.0001	1.52 (1.18, 1.96)	.0011	1.42 (1.10, 1.83)	.0079	1.43 (1.10, 1.85)	.0067
Per 1 SD		1.35 (1.24, 1.46)	<.0001	1.18 (1.07, 1.29)	.0007	1.14 (1.04, 1.26)	.0066	1.14 (1.04, 1.26)	.0072
*P* for trend			<.0001		.0013		.0091		.0076
**Total mortality**									
Ln (100 × UACR)		1.30 (1.27, 1.33)	<.0001	1.27 (1.23, 1.31)	<.0001	1.25 (1.21, 1.29)	<.0001	1.25 (1.21, 1.29)	<.0001
Low	395/3389	Ref		Ref		Ref		Ref	
Middle	602/3391	1.57 (1.38, 1.78)	<.0001	1.45 (1.27, 1.65)	<.0001	1.40 (1.23, 1.60)	<.0001	1.42 (1.24, 1.62)	<.0001
High	937/3391	2.63 (2.34, 2.96)	<.0001	2.16 (1.90, 2.46)	<.0001	2.04 (1.79, 2.32)	<.0001	2.04 (1.79, 2.32)	<.0001
Per 1 SD		1.47 (1.41, 1.53)	<.0001	1.42 (1.36, 1.49)	<.0001	1.39 (1.33, 1.46)	<.0001	1.38 (1.32, 1.45)	<.0001
*P* for trend			<.0001		<.0001		<.0001		<.0001

Model 1: adjusted for age, sex, race, treatment, education, living alone, depression, cardiovascular disease history, family history of heart disease, heart attack, or stroke, heart failure, previous hypertension, duration of diabetes, proteinuria, body mass index, systolic and diastolic blood pressure.

Model 2: adjusted for Model 1 covariables plus HbA1c, total cholesterol, triglycerides, very low-density lipoprotein, low-density lipoprotein cholesterol, high-density lipoprotein cholesterol, alanine aminotransferase, and estimated glomerular filtration rate.

Model 3: adjusted for Model 2 covariables plus the medications use, diuretics, angiotensin receptor blockers/angiotensin converting enzyme inhibitors, calcium channel blockers, beta-blockers, sulfonylureas, biguanides, thiazolidinediones, insulins.

Abbreviations: HR, hazard ratio; UACR, urinary albumin to creatinine ratio.

### Additional Predictive Value of UACR for MACEs and Total Mortality

The predictive value of UACR for MACEs and total mortality was assessed using the AUC, NRI, and IDI (Table 3). Incorporating the UACR into the conventional model significantly enhanced the predictive capability for MACEs and total mortality in participants with T2DM. After incorporating the UACR into the conventional model, there was a significant improvement in the ability to reclassify and differentiate the risks of MACEs, as evidenced by an NRI of 0.126 (95% CI, 0.094–0.157; *P* < .001) and an IDI of 0.012 (95% CI, 0.007–0.017; *P* < .001). Similar findings were observed for total mortality, CVD mortality, nonfatal MI, and nonfatal stroke. These findings suggest that the inclusion of UACR can enhance the predictive efficiency for the risk of MACEs and total mortality in patients with T2DM.

**Table 3.** Additional predictive value of UACR for MACEs and total mortality

	AUC (95% CI)	*P* value	NRI (95% CI)	*P* value	IDI (95% CI)	*P* value
**MACEs**						
Conventional model	0.678 (0.664, 0.691)		Ref		Ref	
Conventional model + ln (100 × UACR)	0.695 (0.682, 0.708)	<.0001	0.126 (0.094, 0.157)	<.0001	0.012 (0.007, 0.017)	<.0001
**CVD mortality**						
Conventional model	0.719 (0.699, 0.740)		Ref		Ref	
Conventional model + ln (100 × UACR)	0.748 (0.729, 0.767)	<.0001	0.193 (0.137, 0.239)	<.0001	0.014 (0.008, 0.022)	<.0001
**Nonfatal MI**						
Conventional model	0.663 (0.645, 0.681)		Ref		Ref	
Conventional model + ln (100 × UACR)	0.676 (0.658, 0.694)	<.0001	0.107 (0.071, 0.149)	<.0001	0.005 (0.002, 0.009)	<.0001
**Nonfatal stroke**						
Conventional model	0.631 (0.607, 0.656)		Ref		Ref	
Conventional model + ln (100 × UACR)	0.644 (0.619, 0.668)	.034	0.141 (0.078, 0.189)	<.0001	0.003 (0.001, 0.007)	<.0001
**Total mortality**						
Conventional model	0.705 (0.692, 0.718)		Ref		Ref	
Conventional model + ln (100 × UACR)	0.723 (0.710, 0.736)	<.0001	0.150 (0.120, 0.178)	<.0001	0.018 (0.013, 0.025)	<.0001

Conventional model: age, sex, CVD history, heart failure, previous hypertension, previous hyperlipidemia, duration of diabetes, BMI, HbA1c, TG, VLDL, LDL-C, HDL-C, eGFR, insulins use.

Abbreviations: CVD, cardiovascular disease; IDI, integrated discrimination improvement; MACEs, major adverse cardiovascular events; MI, myocardial infarction; NRI, net reclassification index; UACR, urinary albumin to creatinine ratio.

### Subgroup Analyses

**Figure 3.**
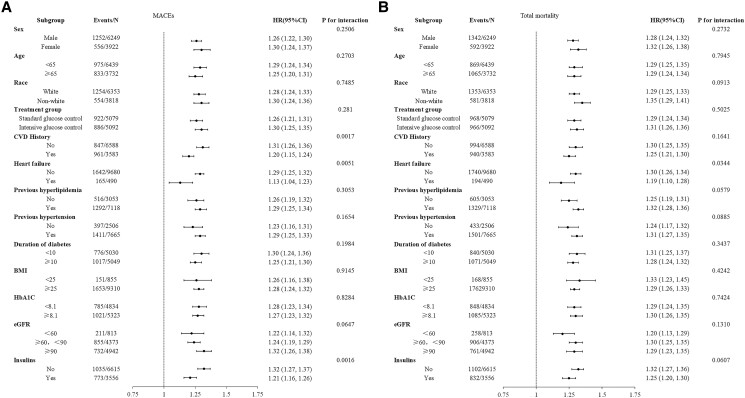
Subgroup and interaction analyses of the association between ln (100 × UACR) and the risk of MACEs and total mortality. A, MACEs; B, total mortality. Participants were stratified by sex, age (<65 years and ≥65 years), race, treatment group (standard glucose control and intensive glucose control), CVD history, heart failure, previous hyperlipidemia, previous hypertension, duration of diabetes (<10 years and ≥10 years), BMI (<25 kg/m^2^ and ≥25 kg/m^2^), HbA1c (<8.1% and ≥8.1%), eGFR (<60 mL/min/1.73 m^2^, ≥60 mL/min/1.73 m^2^, <90 mL/min/1.73 m^2^, and ≥90 mL/min/1.73 m^2^), and insulin use. Non-White participants included individuals of Hispanic, Black, and other ethnic backgrounds. Abbreviations: BMI, body mass index; eGFR, estimated glomerular filtration rate; MACEs, major adverse cardiovascular events; UACR, urinary albumin to creatinine ratio.

To further investigate the association between ln (100 × UACR) and outcome events, subgroup analyses were performed, stratified by sex, age (<65 years and ≥65 years), race, treatment group (standard glucose control and intensive glucose control), CVD history, heart failure, previous hyperlipidemia, previous hypertension, duration of diabetes (<10 years and ≥10 years), BMI (<25 kg/m^2^ and ≥25 kg/m^2^), HbA1c (<8.1% and ≥8.1%), eGFR (<60 mL/min/1.73 m^2^, ≥60 mL/min/1.73 m^2^, <90 mL/min/1.73 m^2^, and ≥90 mL/min/1.73 m^2^), and insulin use in Fig. 3 and elsewhere (18). The findings indicated that a history of CVD, heart failure, and insulin use may contribute to the relationship between ln (100 × UACR) and MACEs. The use of ln (100 × UACR) demonstrated a higher predictive capacity for MACEs in patients with T2DM without a previous medical history of CVD, heart failure, or insulin use. Furthermore, a prior diagnosis of heart failure significantly influenced the relationship between ln (100 × UACR) and total mortality. Similar to MACEs, ln (100 × UACR) was a stronger predictor of total mortality in patients with T2DM without a history of heart failure.

### The Relationship Between ln (100 × UACR) and Outcomes With Normal UACR

**Figure 4.**
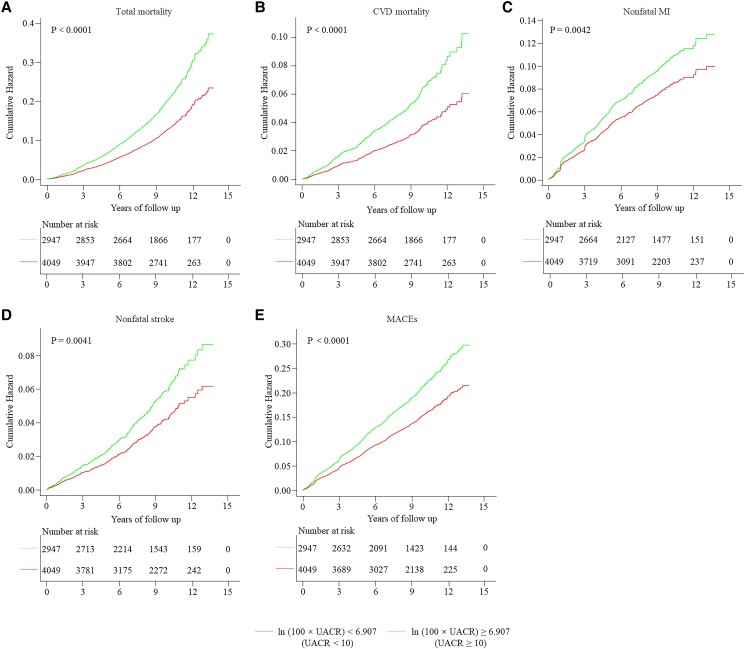
Kaplan-Meier curves for MACEs and total mortality with normal UACR. A, Total mortality; B, CVD mortality; C, Nonfatal MI; D, Nonfatal stroke; E, MACEs. Abbreviations: CVD, cardiovascular disease; MACEs, major adverse cardiovascular events; MI, myocardial infarction; UACR, urinary albumin to creatinine ratio.

Compared to those with low ln (100 × UACR), participants with T2DM with a middle ln (100 × UACR) exhibited a 35% higher risk of MACEs and 42% higher total mortality risk. Moreover, the middle ln (100 × UACR) value was within the normal albuminuria range (UACR <30 mg/g). Consequently, our study was restricted to participants with normal UACR. The 6996 participants with normal UACR were divided into 2 groups based on UACR (UACR < 10 mg/g and UACR ≥10 mg/g). Table 4 shows the baseline characteristics of participants with normal UACR. Participants with a high normal UACR were at an increased risk of MACEs and total mortality. The Kaplan-Meier curves revealed that participants with a high normal UACR had a higher cumulative hazard compared to those with a lower normal UACR in MACEs and total mortality (Fig. 4).

**Table 4.** Baseline characteristics of participants with normal UACR

Characteristics	UACR		*P* value
	<10 mg/g	≥10 mg/g	
N	4049	2947	
UACR (mg/g)	6.00 (4.46–7.66)	15.74 (12.36–20.60)	<.001
Ln (100 × UACR) (mg/g)	6.35 (0.36)	7.39 (0.31)	<.001
Age(years)	62.28 (6.34)	62.97 (6.62)	<.001
Sex, n (%)			.691
Male	2388 (58.98%)	1752 (59.45%)	
Female	1661 (41.02%)	1195 (40.55%)	
Race, n (%)			.273
White	2556 (63.13%)	1898 (64.40%)	
Non-White	1493 (36.87%)	1049 (35.60%)	
Treatment, n (%)			.214
Standard glucose control	2049 (50.61%)	1447 (49.10%)	
Intensive glucose control	2000 (49.39%)	1500 (50.90%)	
Education, n (%)			.008
Less than high school graduate	532 (13.15%)	415 (14.08%)	
High school grad (or GED)	1022 (25.26%)	835 (28.33%)	
Some college or technical school	1372 (33.91%)	930 (31.56%)	
College graduate or more	1120 (27.68%)	767 (26.03%)	
Living alone, n (%)	3273 (80.87%)	2340 (79.40%)	.127
Depression, n (%)	991 (24.49%)	704 (23.89%)	.564
CVD History, n (%)	1226 (30.28%)	1034 (35.09%)	<.001
Family history of heart disease, heart attack, or stroke, n (%)	1917 (49.05%)	1434 (50.49%)	.243
Heart failure, n (%)	143 (3.53%)	148 (5.02%)	.002
Previous hyperlipidemia, n (%)	2845 (70.26%)	2108 (71.53%)	.250
Previous hypertension, n (%)	2917 (72.04%)	2238 (75.94%)	<.001
Duration of diabetes (years)	9.65 (7.15)	10.65 (7.46)	<.001
Proteinuria, n (%)	389 (9.61%)	506 (17.17%)	<.001
BMI (kg/m^2^)	32.08 (5.33)	32.17 (5.32)	.302
SBP (mmHg)	131.89 (15.55)	136.18 (16.34)	<.001
DBP (mmHg)	74.26 (10.03)	74.90 (10.88)	<.012
Heart rate, bpm	72.12 (11.28)	73.03 (11.83)	.002
HbA1C (%)	8.13 (0.98)	8.34 (1.05)	<.001
TC (mg/dL)	181.70 (39.27)	183.84 (41.60)	.083
TG (mg/dL)	176.41 (122.01)	189.21 (136.73)	<.001
VLDL (mg/dL)	34.22 (20.36)	36.49 (23.49)	<.001
LDL-C (mg/dL)	104.72 (32.97)	105.61 (33.51)	.368
HDL-C (mg/dL)	42.75 (11.60)	41.75 (11.32)	<.001
ALT (mg/dL)	27.83 (17.20)	27.84 (15.45)	.320
Serum creatinine (mg/dL)	0.90 (0.21)	0.89 (0.23)	<.061
eGFR (mL/min/1.73 m^2^)	90.98 (23.23)	92.75 (28.21)	.059
Urinary albumin (mg/dL)	0.78 (0.48)	2.08 (1.31)	<.001
Urinary creatinine (g/dL)	0.13 (0.07)	0.12 (0.07)	<.001
Medications, n (%)			
Diuretics	1396 (34.48%)	1044 (35.43%)	.411
ARB/ACEI	2669 (65.92%)	2026 (68.75%)	.013
CCB	622 (15.36%)	514 (17.44%)	.020
Beta-blockers	1039 (25.72%)	892 (30.35%)	<.001
Sulfonylureas	2200 (54.35%)	1600 (54.29%)	.963
Biguanides	2574 (63.59%)	1928 (65.42%)	.113
Meglitinides	111 (2.74%)	73 (2.48%)	.494
Thiazolidinediones	923 (22.80%)	632 (21.45%)	.178
Insulins	1223 (30.20%)	1008 (34.20%)	<.001
Statins	2556 (63.38%)	1895 (64.57%)	.308
Fibrates	245 (6.08%)	196 (6.69%)	.304
Cholesterol absorption inhibitors	81 (2.01%)	61 (2.08%)	.835
MACEs, n (%)	508 (12.55%)	493 (16.73%)	<.001
CVD mortality	141 (3.48%)	170 (5.77%)	<.001
Nonfatal MI	278 (6.87%)	251 (8.52%)	.010
Nonfatal stroke	143 (3.53%)	141 (4.78%)	<.009
Total mortality, n (%)	487 (12.03%)	547 (18.56%)	<.001

Data are shown as mean (SD), median (Q1-Q3), or as n (%). *P* values for the test of the 2 groups of ln (100 × UACR) were obtained by using the χ^2^ test (categorical variables), ANOVA (continuous variables), or Kruskal-Wallis test (nonparametric comparisons).

Abbreviations: ALT, alanine aminotransferase; ARB, angiotensin receptor blocker; ACEI, angiotensin converting enzyme inhibitors; BMI, body mass index; CCB, calcium channel blockers; CVD, cardiovascular disease; DBP, diastolic blood pressure; eGFR, estimated glomerular filtration rate; GED, General Equivalency Diploma; HDL-C, high-density lipoprotein cholesterol; LDL-C, low-density lipoprotein cholesterol; MACEs, major adverse cardiovascular events; MI, myocardial infarction; SBP, systolic blood pressure; TC, total cholesterol; TG, triglycerides; UACR, urinary albumin to creatinine ratio; VLDL, very low-density lipoprotein.

Subsequently, we employed multiple regression analysis to examine the association between UACR and outcome events within the normal range (Table 5). Model 4 was adjusted for age, sex, race, education, living alone, depression, CVD history, family history of heart disease, heart attack, or stroke, heart failure, previous hypertension, duration of diabetes, BMI, SBP, and DBP. Model 5 was further adjusted for HbA1c, TC, TG, VLDL, LDL-C, HDL-C, ALT, and eGFR. Model 6 was adjusted for medication use (diuretics, CCB, beta-blockers, biguanides, thiazolidinediones, and insulins) as an additional covariate for Model 5. In Model 6, there was a significant elevated association between normal UACR and the cumulative risk of MACEs, even after comprehensive adjustment for potential confounding factors (HR 1.23; 95% CI, 1.08–1.40, *P* = .0022). An association was also observed between normal UACR and CVD mortality as well as total mortality. However, no significant association was found with nonfatal MI or nonfatal stroke (Table 5).

**Table 5.** Risk of MACEs and total mortality based on ln (100 × UACR) with normal UACR

Outcome	Events/n	Non-adjusted		Model 4		Model 5		Model 6	
		HR (95% CI)	*P* value	HR (95% CI)	*P* value	HR (95% CI)	*P* value	HR (95% CI)	*P* value
**MACEs**	1001/6996								
Ln (100 × UACR)		1.39 (1.26, 1.54)	<.0001	1.30 (1.17, 1.44)	<.0001	1.27 (1.15, 1.41)	<.0001	1.27 (1.14, 1.41)	<.0001
< 6.907 (UACR < 10)	508/4049	Ref		Ref		Ref		Ref	
≥ 6.907 (UACR ≥ 10)	493/2947	1.39 (1.22, 1.57)	<.0001	1.28 (1.12, 1.45)	.0002	1.24 (1.09, 1.41)	.0014	1.23 (1.08, 1.40)	.0022
Per 1 SD		1.23 (1.15, 1.30)	<.0001	1.18 (1.10, 1.25)	<.0001	1.16 (1.09, 1.24)	<.0001	1.16 (1.08, 1.24)	<.0001
**CVD mortality**	311/6996								
Ln (100 × UACR)		1.69 (1.41, 2.03)	<.0001	1.53 (1.26, 1.85)	<.0001	1.51 (1.24, 1.83)	<.0001	1.50 (1.24, 1.82)	<.0001
< 6.907 (UACR < 10)	141/4049	Ref		Ref		Ref		Ref	
≥ 6.907 (UACR ≥ 10)	170/2947	1.70 (1.36, 2.13)	<.0001	1.53 (1.21, 1.93)	.0004	1.51 (1.19, 1.91)	.0007	1.49 (1.18, 1.89)	.0009
Per 1 SD		1.38 (1.23, 1.55)	<.0001	1.30 (1.15, 1.46)	<.0001	1.29 (1.14, 1.45)	<.0001	1.28 (1.14, 1.45)	<.0001
**Nonfatal MI**	529/6996								
Ln (100 × UACR)		1.29 (1.12, 1.48)	.0004	1.24 (1.07, 1.43)	.0038	1.21 (1.05, 1.40)	.0097	1.21 (1.04, 1.40)	.0122
< 6.907 (UACR < 10)	278/4049	Ref		Ref		Ref		Ref	
≥ 6.907 (UACR ≥ 10)	251/2947	1.28 (1.08, 1.52)	.0043	1.20 (1.01, 1.43)	.0432	1.16 (0.97, 1.39)	.0988	1.16 (0.97, 1.39)	.1083
Per 1 SD		1.17 (1.07, 1.27)	.0004	1.14 (1.04, 1.25)	.0038	1.13 (1.03, 1.23)	.0097	1.12 (1.03, 1.23)	.0122
**Nonfatal stroke**	284/6996								
Ln (100 × UACR)		1.39 (1.15, 1.68)	.0006	1.24 (1.02, 1.50)	.0323	1.19 (0.98, 1.45)	.0873	1.19 (0.98, 1.45)	.0823
< 6.907 (UACR < 10)	143/4049	Ref		Ref		Ref		Ref	
≥ 6.907 (UACR ≥ 10)	141/2947	1.40 (1.11, 1.77)	.0043	1.22 (0.96, 1.54)	.1106	1.15 (0.90, 1.46)	.2629	1.14 (0.90, 1.45)	.2864
Per 1 SD		1.23 (1.09, 1.38)	.0006	1.14 (1.01, 1.29)	.0323	1.11 (0.98, 1.26)	.0873	1.11 (0.99, 1.26)	.0823
**Total mortality**	1034/6996								
Ln (100 × UACR)		1.50 (1.36, 1.66)	<.0001	1.38 (1.25, 1.53)	<.0001	1.35 (1.21, 1.50)	<.0001	1.36 (1.22, 1.51)	<.0001
< 6.907 (UACR < 10)	487/4049	Ref		Ref		Ref		Ref	
≥ 6.907 (UACR ≥ 10)	547/2947	1.59 (1.41, 1.80)	<.0001	1.46 (1.29, 1.66)	<.0001	1.43 (1.25, 1.62)	<.0001	1.44 (1.26, 1.63)	<.0001
Per 1 SD		1.28 (1.21, 1.37)	<.0001	1.22 (1.15, 1.30)	<.0001	1.20 (1.13, 1.28)	<.0001	1.21 (1.13, 1.29)	<.0001

Model 4: adjusted for age, sex, race, education, living alone, depression, CVD history, family history of heart disease, heart attack, or stroke, heart failure, previous hypertension, duration of diabetes, BMI, SBP, DBP.

Model 5: adjusted for Model 4 covariables plus HbA1c, TC, TG, VLDL, LDL-C, HDL-C, ALT, eGFR.

Model 6: adjusted for Model 5 covariables plus medications used, diuretics, CCB, beta-blockers, biguanides, thiazolidinediones, insulins.

Abbreviations: HR, hazard ratio; UACR, urinary albumin to creatinine ratio.

**Figure 5.**
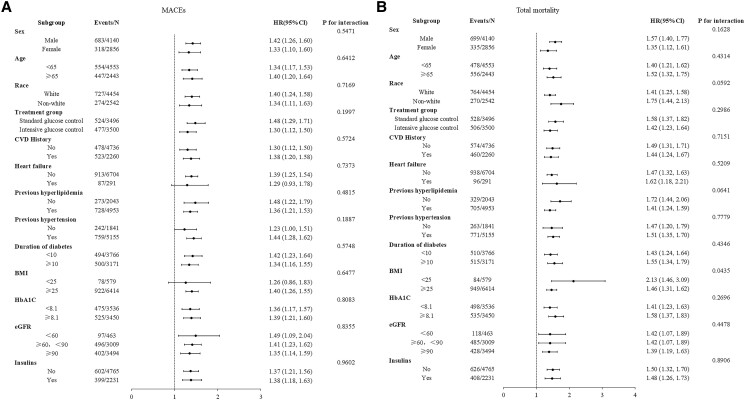
Subgroup and interaction analyses of the association between ln (100 × UACR) and the risk of MACEs and total mortality with normal UACR. A, MACEs; B, total mortality. Participants were stratified by sex, age (<65 years and ≥65 years), race, treatment group (standard glucose control and intensive glucose control), CVD history, heart failure, previous hyperlipidemia, previous hypertension, duration of diabetes (<10 years and ≥10 years), BMI (<25 kg/m^2^ and ≥25 kg/m^2^), HbA1c (<8.1% and ≥8.1%), eGFR (<60 mL/min/1.73 m^2^, ≥60 mL/min/1.73 m^2^, <90 mL/min/1.73 m^2^, and ≥90 mL/min/1.73 m^2^), and insulin use. Non-White participants included individuals of Hispanic, Black, and other ethnic backgrounds. Abbreviations: BMI, body mass index; eGFR, estimated glomerular filtration rate; MACEs, major adverse cardiovascular events; UACR, urinary albumin to creatinine ratio.

To further investigate the association between normal UACR and outcome events, we conducted subgroup analyses stratified by sex, age, race, treatment group, CVD history, heart failure, previous hyperlipidemia, previous hypertension, duration of diabetes, BMI, HbA1c, eGFR, and insulin use in Fig. 5 and elsewhere (18). In patients with T2DM with lower BMI, a higher normal UACR demonstrated a stronger predictive value for total mortality compared to a lower normal UACR.

These findings suggest that patients with T2DM were at a higher risk of MACEs and total mortality, even when UACR was elevated within the normal range.

## Discussion

Of the 10 171 participants with T2DM, a significant association was found between UACR and the risk of MACEs and total mortality in patients with T2DM. Similar associations were also observed for cardiovascular disease mortality, nonfatal MI, and nonfatal stroke.

A comparison of the baseline characteristics of the tertiles in the present study showed that participants with higher UACR had higher SBP, DBP, HbA1c, TG, and VLDL and lower HDL. Our results showed that the UACR can be a valuable indicator for predicting cardiovascular outcomes and enhancing risk stratification. Therefore, early assessment of the UACR in patients with T2DM is important. Furthermore, the inclusion of the UACR in the conventional model (including traditional risk factors) significantly enhanced the predictive capacity for MACEs and total mortality among participants with T2DM.

Albuminuria is recognized as a reliable indicator of systemic endothelial dysfunction (19) and is considered a sensitive prognostic marker for assessing an elevated risk of CVD (20, 21). The UACR is the recommended method for detecting albuminuria (22). The American Diabetes Association recommends that patients with T2DM undergo a UACR test at least annually to identify those at high risk of experiencing severe outcomes (23). Previous studies have shown that patients with T2DM and a higher UACR are at an increased risk of mortality, MACEs, heart failure, and peripheral neuropathy (12, 24, 25). Our study had similar findings. After adjusting for several potential confounding factors, for each 1 SD increase in ln (100 × UACR), participants had a 29% higher risk of developing MACEs and a 38% higher total mortality risk. Previous studies have demonstrated a positive association between microalbuminuria (UACR, 30–300 mg/g) or macroalbuminuria (UACR > 300 mg/g) and an elevated risk of total mortality and cardiovascular disease (26, 27). Our study further revealed that patients with T2DM had an increased risk of total mortality and MACEs even when they exhibited a high normal UACR. We categorized ln (100 × UACR) into 3 equal tertiles: the tertile ranges of ln (100 × UACR) were low (4.27–6.73 mg/g), middle (6.73–7.88 mg/g), and high (7.88–14.07 mg/g), and the corresponding UACRs were low (0.72–8.33), middle (8.33–26.52), and high (26.52–12 908.16), respectively. The middle ln (100 × UACR) value was within the normal albuminuria range (UACR <30 mg/g). Compared to those with low ln (100 × UACR), participants with T2DM with a middle ln (100 × UACR) exhibited a 35% higher risk of MACEs and 42% higher total mortality risk.

A UACR threshold >10 mg/g demonstrated a substantial predictive capacity for both the cumulative incidence and progression of chronic kidney disease in patients with T2DM and UACR within the normal range (28). In addition, The UACR is associated with risk factors for adverse cardiovascular outcomes within the normal range. It is associated with an elevated risk of developing hypertension, T2DM, and dyslipidemia, at levels below the usual threshold for microalbuminuria (29-31). Furthermore, both our and previous studies have demonstrated that the UACR is associated with an increased risk of adverse cardiovascular outcomes, even when it falls within the normal range (32, 33). The UACR within the normal range was associated with an increased risk of cardiovascular disease, and a UACR cutoff of 10 mg/g is considered optimal for diagnosing diabetic left ventricular hypertrophy (34). Patients with T2DM require a revised definition of albuminuria. Our study demonstrated that patients with T2DM with a UACR between 10 and 30 mg/g have a higher risk of MACEs and total mortality compared to those with a UACR less than 10 mg/g. For each 1 SD increase in ln (100 × UACR) with normal UACR, there was a 16% higher risk of participants developing MACEs (HR 1.16; 95% CI, 1.08–1.24, *P* < .0001), and a 21% higher risk of participants developing MACEs (HR 1.21; 95% CI, 1.13–1.29, *P* < .0001). These pieces of evidence challenge the notion that a UACR of less than 30 mg/g indicates “normal” albumin excretion, especially for patients with T2DM.

The existing literature on the interaction between UACR and eGFR with respect to total mortality and MACEs has yielded inconsistent findings (11, 12, 35). No significant interaction was found between the UACR and eGFR with respect to MACEs or total mortality. However, a significant interaction was observed for cardiovascular mortality. In our study, a stronger association was observed between the UACR and total mortality in participants without a history of heart failure. Additionally, a more pronounced association between the UACR and MACEs was found in participants without insulin use or a previous history of CVD or heart failure. One possible explanation for this observation is that participants who used insulin or had a history of CVD or heart failure may have been more proactive in managing their UACR. Previous studies have demonstrated a higher risk of total mortality and cardiovascular disease in women than in men, as indicated by the UACR (36, 37). We also found that compared to men, women had a higher risk of nonfatal MI following the UACR but had a lower risk of nonfatal stroke. The mechanism underlying the sex-specific association between the UACR and long-term adverse cardiovascular outcomes remains unclear and warrants further investigation.

The strengths of this study include the large cohort size and comprehensive follow-up regarding MACEs, including cardiovascular mortality, nonfatal MI, nonfatal stroke, and total mortality. However, this study had some limitations. First, this was a post hoc analysis of the ACCORD and ACCORDION trials. Although we adjusted for potential confounders in the multivariate Cox regression analysis, there may have been some confounders in the original studies that were beyond our control. Second, the participants in the ACCORD and ACCORDION trials were specifically patients with T2DM who were at high risk for CVD events; therefore, further research is needed to determine the impact of the UACR on the risk of adverse cardiovascular outcomes in other populations. Finally, changes in the UACR were not continuously monitored throughout the follow-up period, highlighting the need for additional studies to assess its predictive value for adverse cardiovascular outcomes.

In conclusion, in our post hoc analysis of the ACCORD and ACCORDION trials, we discovered that the UACR in patients with T2DM could predict the risk of developing MACEs and total mortality even when it was within the normal range. This study offers valuable insights into the assessment of MACEs and total mortality risk in patients with T2DM.

## Data Availability

Some or all datasets generated during and/or analyzed during the current study are not publicly available but are available from the corresponding author on reasonable request.

## Clinical Trial Information

ClinicalTrials.gov registration no. NCT00000620.

## Abbreviations

ACCORD: Action to Control Cardiovascular Risk in Diabetes
ACEI: angiotensin converting enzyme inhibitor
ACCORDION: the ACCORD follow-up study
ALT: alanine aminotransferase
ARB: angiotensin receptor blocker
ANOVA: analysis of variance
AUC: area under the curve
BMI: body mass index
CCB: calcium channel blocker
CVD: cardiovascular disease
DBP: diastolic blood pressure
DM: diabetes mellitus
eGFR: estimated glomerular filtration rate
HbA1c: glycated hemoglobin
HDL-C: high-density lipoprotein cholesterol
HR: hazard ratio
IDI: integrated discrimination improvement
LDL-C: low-density lipoprotein cholesterol
MACEs: major adverse cardiovascular events
MI: myocardial infarction
NRI: net reclassification improvement
SBP: systolic blood pressure
T2DM: type 2 diabetes mellitus
TC: total cholesterol
TG: triglycerides
UACR: urinary albumin to creatinine ratio
VLDL: very low-density lipoprotein
